# Testing the specificity of executive functioning impairments in adolescents with ADHD, ODD/CD and ASD

**DOI:** 10.1007/s00787-017-1089-5

**Published:** 2017-12-09

**Authors:** Virginia Carter Leno, Susie Chandler, Pippa White, Andrew Pickles, Gillian Baird, Chris Hobson, Anna B. Smith, Tony Charman, Katya Rubia, Emily Simonoff

**Affiliations:** 1 Institute of Psychiatry, Psychology and Neuroscience, King’s College London, 16 De Crespigny Park, London, SE5 8AF UK; 2 Guy’s and St Thomas’ NHS Foundation Trust, London, UK; 3 Cardiff University, Cardiff, UK

**Keywords:** Attention deficit hyperactivity disorder, Conduct disorder, Autism spectrum disorder, Executive functioning, Cognition

## Abstract

Current diagnostic systems conceptualise attention deficit hyperactivity disorder (ADHD), oppositional defiant/conduct disorder (ODD/CD) and autism spectrum disorder (ASD) as separate diagnoses. However, all three demonstrate executive functioning (EF) impairments. Whether these impairments are trans-diagnostic or disorder-specific remains relatively unexplored. Four groups of 10–16 year-olds [typically developing (TD; *N* = 43), individuals clinically diagnosed with ADHD (*N* = 21), ODD/CD (*N* = 26) and ASD (*N* = 41)] completed Go/NoGo and Switch tasks. Group differences were tested using analysis of co-variance (ANCOVA) including age, IQ, sex, conduct problems and ADHD symptoms as co-variates. Results indicated some disorder-specificity as only the ASD group demonstrated decreased probability of inhibition in the Go/NoGo task compared to all other groups. However, shared impairments were also found; all three diagnostic groups demonstrated increased reaction time variability (RTV) compared to the TD group, and both the ODD/CD and the ASD group demonstrated increased premature responses. When controlling for ADHD symptoms and conduct problems, group differences in RTV were no longer significant; however, the ASD group continued to demonstrate increased premature responses. No group differences were found in cognitive flexibility in the Switch task. A more varied response style was present across all clinical groups, although this appeared to be accounted for by sub-threshold ODD/CD and ADHD symptoms. Only the ASD group was impaired in response inhibition and premature responsiveness relative to TD adolescents. The findings suggest that some EF impairments typically associated with ADHD may also be found in individuals with ASD.

**Electronic supplementary material:**

The online version of this article (10.1007/s00787-017-1089-5) contains supplementary material, which is available to authorized users.

## Introduction

Current diagnostic systems conceptualise attention deficit hyperactivity disorder (ADHD), oppositional defiant/conduct disorder (ODD/CD) and autism spectrum disorder (ASD) as distinct diagnoses. ADHD is characterised by persistent symptoms of age-inappropriate inattention, hyperactivity and impulsivity; ODD by negative, hostile and defiant behaviour; CD by aggression, destruction of property and serious violations of rules; and ASD by impairments in social communication abilities and the presence of restricted and repetitive behaviours and interests and sensory anomalies [1].

Traditional neuropsychological approaches posit that psychiatric disorders are underpinned by impairments in specific domains of functioning, known as intermediate phenotypes, and said phenotypes discriminate between diagnostic categories. These phenotypes may represent potential risk factors and targets for intervention. However, some have highlighted that the search for discriminative phenotypes has not been as successful as hoped [2], and suggest a research framework that disregards diagnostic categories and focuses instead upon continuous associations between brain functioning and symptomatology (Research Domain Criteria; [2]). Although some report shared impairments between the diagnostic groups [3–7], prior work has not consistently accounted for high rates of co-occurrence between these disorders, which is widely reported (e.g., [8–10]). Therefore, prior findings may in part reflect unacknowledged co-morbidity. Aetiologically, it is crucial to understand whether EF impairments are indicative of psychopathology in general or differentiate between diagnostic categories. The current study aimed to examine EF impairments among ADHD, ODD/CD and ASD groups, whilst controlling for co-occurring ADHD and ODD/CD symptoms.

In terms of distinct cognitive profiles, increased impulsivity, indexed by both impairments in response inhibition and a premature response style, is thought to be characteristic of individuals with ADHD [11, 12]. However, impairments are also reported in ODD/CD [4, 13] and ASD [14]. Similarly, although cognitive flexibility impairments are most characteristic of ASD [15], impairments are also reported in ADHD and ODD/CD [16]. Additionally, all three disorders demonstrate increased intra-subject response time variability (RTV) [16, 17]. Despite this overlap in EF impairments, comparisons between disorders are limited and inconsistent.

Some comparative studies of inhibition between individuals with ADHD and those with ASD found that only those with ADHD showed impairment [18–20]; however, others failed to find group differences [3, 21], and one study found that the ASD group showed greatest impairment [22]. A meta-analysis found that both ADHD and ODD/CD were independently characterised by inhibition impairment [6], and comparison of individuals with ODD/CD with or without ADHD found that both groups showed slower stop signal reaction time on the Stop task; however, only the ODD/CD + ADHD group was impaired in motor inhibition in the Go/NoGo task [4].

Some studies that compared ADHD and ASD groups on cognitive flexibility found that only the ASD group showed impairment [3, 19], while others have failed to find group differences [18, 21]. Studies have not found evidence of dissociation in cognitive flexibility impairment between ADHD and ODD/CD [4, 23].

In the third area of potential overlap, RTV, studies have found increased RTV in ADHD and ASD + ADHD groups but not in ASD alone [24], whereas some report increased RTV in ASD but not ADHD [7, 25]. Both continuous analyses of symptoms and group-based comparisons found that both ADHD and ODD/CD were characterised by increased premature responses and RTV [4, 26].

To our knowledge, no study has directly compared EF among adolescents with ADHD, ODD/CD and ASD. Furthermore, many prior comparative studies have not controlled for co-occurring symptoms. We compared three disorders, ADHD, ODD/CD and ASD, whilst controlling for conduct problems and ADHD symptoms. We also used a more representative sample of young people with ASD (IQ range of 54–129), as most studies only include those with an IQ ≥ 70, yet around half of children with ASD have an IQ < 70 [27]. Informed by the prior literature described above, we tested group differences in response inhibition (Go/NoGo task) and cognitive flexibility (Switch task). We also tested premature responses and RTV across both tasks. We hypothesised that all clinical groups would be characterised by impairments in response inhibition with most severe impairments in ADHD [16] while the ASD group only would show impairments in cognitive flexibility [3]. Additionally, we hypothesised that increased premature responses would be more typical of ADHD, while increased RTV would be observed in all groups [4, 17].

## Method

### Sample

Across the ODD/CD, ADHD and typically developing (TD) groups only those aged 10–16 years were selected from original samples, which had a wider age range. This was to encompass a similar range to the ASD group. All participants had information on ADHD and ODD/CD symptoms, along with measures of neurocognitive task performance. Due to the post hoc nature of the current study, information was not available on ASD symptoms in the ADHD, ODD/CD and TD groups. The ODD/CD group, along with part of the TD and ADHD groups included participants from a larger study contrasting ODD/CD and ADHD (see Hobson et al. [4] for full details). The remainder of the TD and ADHD participants came from a different study exploring EF impairments in ADHD (see Rubia et al. [12] for full details). The ODD/CD, ADHD and ASD samples were all recruited through clinics. Informed consent was obtained for all participants. Ethical approval was obtained for each study, in accordance with the ethical standards laid down in the 1964 Declaration of Helsinki, from which samples were drawn for current analysis.

### ODD/CD group (*n* = 26)

Adolescents were recruited from two existing longitudinal samples in which participants had been clinically referred for oppositional problems in childhood [29, 30]. To confirm ODD/CD, parents were interviewed using the ODD/CD sections of the Child and Adolescent Psychiatric Assessment (CAPA; [28]). Participants were not included in the ODD/CD group if they met criteria for ADHD, or if they had ever received a clinical diagnosis of ADHD, or if they had ever received a clinical diagnosis of ASD.

### ADHD group (*n* = 21)

Participants from Hobson et al. [4] who met the criteria for ADHD but not ODD/CD formed part of the ADHD group (*n* = 9). Participants had to have symptoms meeting ADHD criteria in at least one domain (i.e., home or school), and demonstrate ‘some impairment’ (defined here as above a 20% cut-off based on age-related published norms) in the other domain on the Conners’ ADHD Parent and Teacher Scales [31]. Individuals were classified as meeting criteria if respondents endorsed at least six of the inattentive or hyperactive/impulsive items. Participants were also included in the ADHD group if they had a current clinical diagnosis of ADHD. In the same manner as the ODD/CD group, ADHD participants from the Hobson et al. sample were not included if they had a clinical diagnosis of ASD. The remainder (*n* = 12) of participants from the Rubia et al. sample [12] had a clinical diagnosis of hyperkinetic disorder (using ICD-10) and met DSM-IV criteria for ADHD-combined type as assessed by an experienced child psychiatrist using a standardised diagnostic interview [32]. The assessment process also included information from other sources (e.g., parents and teachers), developmental history, and behavioural observation of the child. Participants were excluded if they had another psychiatric disorder (including ODD/CD and ASD), neurological abnormalities or epilepsy. Participants taking stimulants were medication-free for at least 18 h prior to testing. Participants with ADHD taken from the Hobson et al. [4] sample vs. the Rubia et al. [12] sample did not differ in age, IQ or severity of ADHD symptoms (all *p*s > 0.05).

### ASD group (*n* = 41)

Participants were part of QUEST, a longitudinal sample in which participants had been clinically referred for ASD-related difficulties in childhood [33]. All participants had a clinical diagnosis of ASD by age 4 years and the “intensively studied” group included at present had their diagnosis confirmed at age 10–16 years with the Autism Diagnostic Observation Schedule (ADOS; [34]) and the Autism Diagnostic Interview-Revised (ADI-R; [35]). All participants were above threshold on either or both the ADOS and the ADI. The “intensively studied” sample was selected to over-represent females, as the main aims of the study included sex comparisons. From the original QUEST sample (which had an IQ range of 19–120), only those who were able to complete the neurocognitive tasks were included in present analyses. Participants were excluded if they scored above the population-defined cut-off of ≥ 4 on the conduct problems sub-scale on the Strengths and Difficulties Questionnaire (SDQ; [36]) (*n* = 4). Those who were above threshold on the SDQ ADHD symptoms sub-scale cut-off of ≥ 7 (*n* = 9) were retained in sensitivity analyses.

### TD group (*n* = 43)

The TD group was a combination of participants from Hobson et al. [4] (*n* = 32) and Rubia et al. [12] (*n* = 11). This consisted of healthy adolescents with no history of, or current psychiatric disorder or intellectual disability, and who fell below cut-off on the SDQ hyperactivity and conduct sub-scales in the Rubia et al. [12] sample, and did not meet diagnostic thresholds on the Conners’ or CAPA in the Hobson et al. [4] sample.

### Questionnaires

The parent-rated SDQ [37] was used to measure psychiatric symptoms. The SDQ comprises three psychiatric sub-scales of ADHD symptoms, conduct, and emotional problems, along with further sub-scales of peer-relationship problems and prosocial behaviour. Current analyses used the ADHD symptoms and conduct problems sub-scales.

### Neurocognitive assessment

All participants completed two tasks selected from the computerised Maudsley Attention and Response Suppression task battery (MARS; [12]). All researchers administering the tasks were trained by the battery developer (KR).

### Go/NoGo task

This task measures selective motor response inhibition. A motor response has to be executed when green space ships appear (go trials; 74%) and inhibited when red enemy planets appear (no-go trials; 26%). The task contains two blocks of right-handed and left-handed responses, respectively, to go trials. The dependent variable is the percentage of successfully inhibited no-go trials (probability of inhibition).

### Switch task

This task measures visual–spatial attention shifting between two spatial dimensions. Participants observe a grid divided into four squares, in the centre of which is a double-headed arrow which switches between horizontal and vertical dimensions. Red dots appear one-by-one in any of the four corners of the grid. When the arrow is horizontal, participants are asked to press the left or right button according to the location of the dot; when the arrow is vertical, participants press either the top or bottom button. The switch from the vertical to the horizontal dimensions appeared in 29% of trials. The main dependent variables are the switch error and reaction time costs (mean errors/reaction times to switch trials—mean errors/reaction times to repeat trials).

### Premature responses and intra-individual response variability

For both tasks, percentage of premature responses, thought to measure an impulsive response style, as responses were made before stimuli have been processed (i.e., responses made 200 ms before and 100 ms after stimulus onset; [12]) and the intra-individual coefficient of variability (ICV) (standard deviation (SD)/mean RT of responses × 100; [12]) were calculated. The distribution of premature responses was positively skewed, thus transformed into a binary variable for both tasks (no premature responses = 0, any premature responses = 1).

### Cognitive ability

Cognitive ability was estimated using the Wechsler Abbreviated Scale of Intelligence (WASI; [37]). Where necessary because of low intellectual ability in the ASD sample (*n* = 2) the Wechsler Preschool and Primary Scale of Intelligence (WPPSI-IV; [38]) was used and a developmental quotient (DQ) calculated. A sub-set of ADHD participants (*n* = 7) were assessed using Raven’s Standard Progressive Matrices Intelligence Questionnaire [39], and scores were converted to estimated IQs on the basis of a series of Ravens-IQ extrapolations performed on larger datasets, by Lord 1988 (unpublished).

### Statistical analyses

Analysis was undertaken in Stata 11. Variables were transformed where necessary (probability of inhibition using square root, ICV for the Go/NoGo task using Box-Cox). Univariate ANOVAs first tested unadjusted group differences. Next, ANCOVA tested group differences adjusted for age, IQ and sex. This ANCOVA was our primary contrast. For the binary premature response variables, logistic regression followed by the Wald test was used. Following this, SDQ conduct problems and ADHD symptoms were separately controlled for, in addition to age, IQ and sex, to explore the influences of sub-threshold traits upon any significant group differences in the adjusted ANCOVA. Two separate sensitivity analyses were conducted (1) excluding participants with IQ < 70 (*N* = 9) and (2) excluding ASD participants scoring above the SDQ ADHD symptom sub-scale (*N* = 9). Where group differences were found in our primary contrast, subsequent unadjusted and adjusted post hoc group contrasts were also performed (adjusting for age, IQ, sex). Exploratory adjusted post hoc contrasts were also conducted, separately adjusting for ADHD symptoms and conduct problems, in addition to age, IQ and sex. The details of all post hoc contrasts are presented in the Supplementary Appendix. The effect sizes of diagnostic group status were calculated using partial *η* ^2^ for continuous variables, and *w* for binary variables [40].

## Results

Table 1 shows group demographics. The ASD group was older than all other groups (*p*s < 0.05), and had lower IQ than the TD and ODD/CD groups (*p*s < 0.01). The ADHD and TD groups had a higher percentage of male participants than the ODD/CD and ASD groups (*p*s < 0.05).

**Table 1:** Sample demographics

Mean (SD; range)	TD (*n* = 43)	ADHD (*n* = 21)	ODD/CD (*n* = 26)	ASD (*n* = 41)	Group differences
Age	12.79 (1.61; 10.17–16)	12.98 (1.47; 10.50–15.75)	12.31 (1.62; 10.20–15.51)	13.77 (1.08; 11.33–15.67)	ASD > TD*, ODD/CD**
IQ	104.95 (11.67; 75–130)	95.29 (13.26; 69–120)	101.42 (14.68; 72–130)	88.49 (19.71; 54–129)	TD > ASD**, ADHD** ODD/CD > ASD**
% male	83.72%	95.24%	65.38%	58.54%	ADHD > ADHD**, ODD/CD* TD > ASD*
SDQ hyperactivity	2.53 (1.61; 0–6)	7.95 (2.04; 3–10)	6.58 (2.23; 1–10)	4.62 (2.49; 0–9)	ADHD, ODD/CD, ASD > TD** ADHD > ODD/CD*, ASD** ODD/CD > ASD**
SDQ conduct problems	1.16 (1.04; 0–3)	3.58 (1.54; 1–7)	5 (1.83; 1–8)	1.41 (1.12; 0–3)	ADHD, ODD/CD > TD** ODD/CD > ADHD**, ASD** ADHD > ASD**

*SDQ* Strengths and Difficulties Questionnaire

** *p* < 0.01, * *p* < 0.05

### Neurocognitive performance

#### Inhibition

Table 2 details task performance by group and Table 3 details the effect size of diagnostic group comparisons in each analysis.

**Table 2:** Group performance on Go/NoGo and Switch task

Mean (SD; range)	TD (*n* = 42)	ADHD (*n* = 21)	ODD/CD (*n* = 26)	ASD (*n* = 41, *n* = 37 for Switch task)
Go/NoGo: probability of inhibition	84.33 (13.18; 28–100)	78.67 (15.86; 38–96)	81.62 (12.39; 50–100)	65.27 (18.76; 20–92)
Switch: reaction time cost	42.61 (43.87; − 29.86–160.40)	40.78 (53.37; − 34.27–199.98)	66.80 (66.10; − 114.44–230.65)	42.23 (56.82; − 74.01–199.99)
Switch: error cost	4.56 (7.44; − 8.29–29.14)	4.65 (8.28; − 5.08–18.56)	5.76 (9.22; − 9.47–27.53)	3.44 (7.01; − 3.39–28.78)
Go/NoGo: premature responses	23.81%	47.62%	50%	70.73%
Switch: premature responses	4.88%	28.57%	34.62%	21.62%
Go/NoGo: ICV	22.94 (6.48; 13.93–42.10)	28.75 (7.89; 16.54–48.37)	26.70 (4.21; 17.31–32.93)	31. 51 (12.54; 16.58–60.90)
Switch: ICV	24.68 (5.63; 16.98–36.48)	28.61 (6.49; 18.13–40.57)	30.18 (5.04; 19.99–42.57)	27.74 (5.51; 15.93–39.54)

*ICV* intra-individual coefficient of variation

**Table 3:** Effect size of diagnostic group in un/adjusted tests of group means and sensitivity analyses

	Co-variation analyses				Sensitivity analyses		
	Unadjusted group differences	Adjusted for IQ, age, sex	Adjusted for ADHD, IQ, age, sex	Adjusted for conduct problems, IQ, age, sex	Exclude IQ < 70	Exclude those in ASD over ADHD cut-off	Post hoc contrasts of adjusted group means (adjusted for IQ, age, sex)
Effect size as indicated by partial η^2^							
Go/NoGo: probability of inhibition	0.23**	0.21**	0.21**	0.21**	0.19**	0.17**	ASD < CD/ODD**, ADHD**, TD**
Switch: reaction time cost	0.03	0.04	0.04	0.02	0.04	0.04	–
Switch: error cost	0.01	0.02	0.02	0.02	0.01	0.02	–
Go/NoGo: ICV	0.15**	0.10**	0.05	0.08*	0.13**	0.13**	TD < ADHD*, ODD/CD*, ASD**
Switch: ICV	0.12**	0.09**	0.04	0.02	0.13**	0.13**	TD < ADHD*, ODD/CD**
Effect size as indicated by *w*							
Go/NoGo: premature responses	0.36**	0.31**	0.27*	0.30**	0.33**	0.32**	ODD/CD > TD^ ASD > TD**
Switch: premature responses	0.26*	0.23	0.17	0.21	0.26*	0.27*	–

*ICV* intra-individual coefficient of variability

** *p* < 0.01, * *p* < 0.05, ^ *p* = 0.06; for partial *η* ^2^, 0.1 = small, 0.6 = medium, 0.14 = large effect; for *w*, 0.1 = small, 0.3 = medium, 0.5 = large effect [40]

Group differences were found in probability of inhibition [*F*(3,126) = 12.84, *p* < 0.01]. These remained when controlling for age, IQ and sex [*F*(3, 123) = 10.76, *p* < 0.01], ADHD symptoms, age, IQ and sex [*F*(3, 118) = 10.33, *p* < 0.01], and conduct problems, age, IQ and sex [*F*(3, 116) = 10.29, *p* < 0.01].

**Fig. 1:**
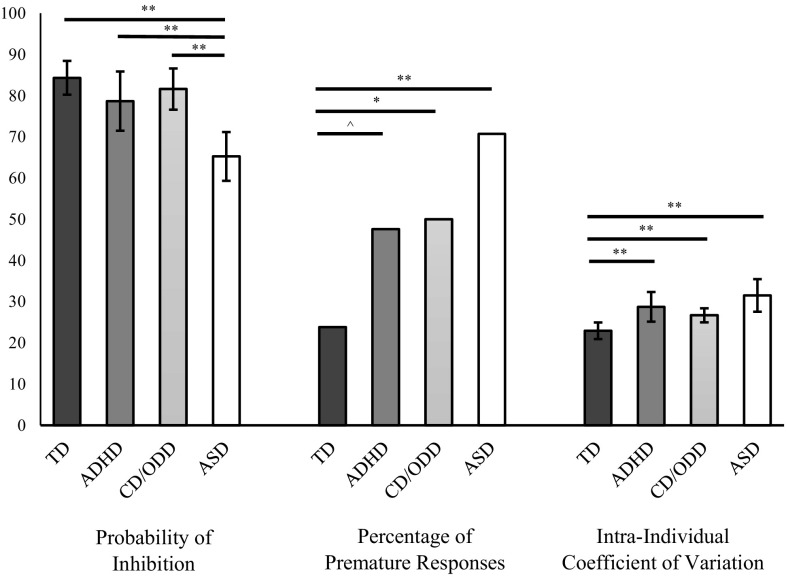
Unadjusted group performance on the Go/NoGo task. **p < 0.01, *p < 0.05, ^*p* = 0.06

Results remained significant in sensitivity analyses excluding those with IQ < 70 [*F*(3, 117) = 9.40, *p* < 0.01], and excluding those who scored above ADHD threshold in the ASD group [*F*(3, 117) = 8.06, *p* < 0.01]. Unadjusted post hoc contrasts found that the ASD group had a lower probability of inhibition than the TD, ADHD and ODD/CD groups (all *p*s < 0.01; Fig. 1). Adjusted post hoc contrasts found a comparable pattern of results in terms of the ASD group having a lower probability of inhibition than all other groups when all co-variates were controlled for (all *p*s < 0.05; Table 2).

### Cognitive flexibility

No group differences were found in the Switch task, for either RT [*F*(3, 121) = 1.38, *p* = 0.25], or error [*F*(3, 121) = 0.45, *p* = 0.72] costs. This remained when controlling for possible confounders and in sensitivity analyses.

### Premature responses

Differences were found in the proportion of participants in each group who demonstrated premature responses on the Go/NoGo task [*Χ* ^2^(3) = 16.84, *p* < 0.01]. These remained when controlling for age, IQ and sex [*Χ* ^2^(3) = 12.45, *p* < 0.01], ADHD symptoms, age, IQ and sex [*Χ* ^2^(3) = 9.54, *p* < 0.05], and conduct problems, age, IQ and sex [*Χ* ^2^(3) = 11.58, *p* < 0.01].

Results remained significant in sensitivity analyses excluding those with IQ < 70 [*X* ^2^ (3) = 13.17, *p* < 0.01], and excluding those who scored above ADHD threshold in the ASD group [*Χ* ^2^(3) = 12.54, *p* < 0.01]. Unadjusted post hoc contrasts found that the ASD (*p* < 0.01) and ODD/CD (*p* < 0.05) groups had a higher proportion of individuals showing premature responses than the TD group. The ADHD vs. TD contrast was significant at trend level (*p* = 0.06). The clinical groups were not significantly different from each other (Fig. 1). Post hoc contrasts adjusted for age, sex and IQ found the ASD group had a higher proportion of individuals showing premature responses than the TD group (*p* < 0.01). The ODD/CD vs. TD contrast was at a trend level (*p* = 0.06). The ADHD vs. TD contrast became non-significant. Only the ASD vs. TD contrast remained significant when controlling for ADHD symptoms, age, IQ and sex (*p* < 0.05) and when controlling for conduct problems, age, IQ and sex (*p* < 0.01).

Differences were also found in the proportion of participants in each group who demonstrated premature responses on the Switch task [*Χ* ^2^(3) = 8.21, *p* < 0.05], but dropped to a trend level when controlling for age, IQ and sex [*Χ* ^2^(3) = 6.75, *p* = 0.08], but became non-significant when controlling for ADHD symptoms, age, IQ and sex [*Χ* ^2^(3) = 3.53, *p* = 0.32], and conduct problems, age, IQ and sex [*Χ* ^2^(3) = 5.51, *p* = 0.14]. Group differences were significant in sensitivity analyses excluding those with IQ < 70 [*Χ* ^2^(3) = 8.02, *p* < 0.05] and excluding those who scored above ADHD threshold in the ASD group [*Χ* ^2^(3) = 8.61, *p* < 0.05].

### Intra-individual response variability

Group differences in ICV were found on the Go/NoGo task [*F*(3, 126) = 6.84, *p* < 0.01]. These remained when controlling for age, IQ and sex [*F*(3, 123) = 4.47, *p* < 0.01], and conduct problems, age, IQ and sex [*F*(3, 116) = 3.34, *p* < 0.05]. Controlling for ADHD symptoms, age, IQ and sex resulted in findings losing significance [*F*(3, 118) = 1.86, *p* = 0.14]. Differences remained significant in sensitivity analyses excluding those with IQ < 70 [*F*(3, 117) = 5.58, *p* < 0.01], and excluding those who scored above ADHD threshold in the ASD group [*F*(3, 117) = 5.75, *p* < 0.01].

Group differences in ICV were also found on the Switch task [*F*(3, 121) = 5.67, *p* < 0.01], and remained when controlling for age, IQ and sex [*F*(3, 118) = 3.99, *p* < 0.01]. Group differences became non-significant when controlling for ADHD symptoms, age, IQ and sex [*F*(3, 114) = 1.41, *p* = 0.24], and conduct problems, age, IQ, and sex [*F*(3, 112) = 0.61, *p* = 0.61]. Sensitivity analyses showed that differences remained significant when excluding those with IQ < 70 [*F*(3, 113) = 5.53, *p* < 0.01] and excluding those who scored above ADHD threshold in the ASD group [*F*(3, 112) = 5.57, *p* < 0.01].

In both tasks unadjusted post hoc contrasts found that all clinical groups had higher ICV than the TD group (all *p*s < 0.01). In the Go/NoGo task the clinical groups were not significantly different from each other (Fig. 1), whereas in the Switch task the ODD/CD group had higher ICV than the ASD group (*p* < 0.05).

In the Go/NoGo task adjusted post hoc contrasts showed that all three clinical groups had significantly higher ICV when controlling for age, IQ and sex (all *p*s < 0.05). When controlling for ADHD symptoms, age, IQ and sex, and then conduct problems, age, IQ and sex, only the ASD group had higher ICV than the TD group (*p* < 0.05 and *p* < 0.01, respectively). In the Switch task adjusted post hoc contrasts found that only the ADHD group (*p* < 0.05) and the ODD/CD group (*p* < 0.01) had significantly higher ICV than the TD group when controlling for age, IQ and sex. The post hoc contrast between the ODD/CD group remained at trend level when controlling for ADHD symptoms, age, IQ and sex (*p* = 0.05). The post hoc contrast between the ADHD group and the TD group was not significant when controlling for conduct problems, age, IQ and sex.

## Discussion

There is on-going debate as to whether diagnostic categories are associated with specific or shared cognitive phenotypes. The current study compared EF performance between ADHD, ODD/CD, ASD and TD groups, whilst controlling for co-occurring ADHD and ODD/CD symptoms. Results indicate shared impairments in some performance measures; all three clinical groups demonstrated increased RTV; although co-variation analyses suggested that this might in part be due to co-occurring ADHD and ODD/CD symptoms. Additionally, both the ASD and the ODD/CD group showed increased premature responses, although only the ASD continued to show impairment when co-occurring ADHD symptoms were controlled for. Results also found disorder-specific impairments, in that only the ASD group showed impairment in inhibition in the Go/NoGo task, relative to the TD group. Contrary to our hypothesis, the ASD group did not show specific impairments in cognitive flexibility. Results suggest that some EF impairments previously thought to be more characteristic of ADHD, such as increased premature responding and RTV, and impaired response inhibition, may also be present in other disorders, such as ASD.

A more premature-impulsive and variable response style is typically attributed to ADHD [12]. Our findings, however, suggest that this may also be found in ODD/CD and ASD, although it is possible that co-occurring ADHD symptoms influenced impairments in the ODD/CD group. Although all three clinical groups demonstrated increased premature responses on the Go/NoGo task, only the ASD-TD and ODD/CD-TD contrasts remained significant, or at trend, when co-varying for IQ, age and sex. Further exploratory adjusted post hoc contrasts suggested that ADHD symptoms may be in part driving the increased level of premature responses in the ODD/CD group, as when ADHD symptoms, IQ, age and sex were controlled for, the ODD/CD vs. TD group contrast became non-significant. This was not the case for the ASD group, who had significantly higher levels of premature response than the TD group, even when ADHD symptoms and conduct problems were controlled for. In terms of the ADHD group, although the unadjusted post hoc contrast between the ADHD and TD group was at a trend level, the contrast adjusted for age, IQ and sex was non-significant. Thus, differences between the ADHD and the TD group in age, IQ and gender may have contributed to significant results in the unadjusted contrast. However, given that in the original sample, those with ADHD had increased premature responses [4], we speculate that by selecting a smaller sub-sample of ADHD cases (*n* = 21), within a specific age range, we limited our statistical power to detect significant effects.

All three clinical groups also demonstrated increased RTV in the Go/NoGo task, in agreement with prior literature [4, 17, 25]. However, when ADHD and ODD/CD symptoms were controlled for, the effect of group mostly lost significance. This suggests that increased RTV may have been in part accounted for by sub-threshold ADHD and ODD/CD symptoms, but was not related to ASD status. This is in line with findings that within those with ASD, only those with co-occurring ADHD show increased RTV [24]. Interestingly, on the Switch task only the ADHD and ODD/CD groups demonstrated increased RTV, whereas the ASD group did not demonstrate any differences in RTV as compared to the TD group. As the Switch task could be seen as a slower task, in that it has longer stimuli presentation times than the Go/NoGo task, and speed of response was not stressed in this task, this could explain differences in RTV between the two tasks in the ASD group. Overall, results regarding RTV suggest that although increased RTV is found across diagnostic categories [16, 17], it may be a marker of underlying symptoms, rather than a shared cognitive phenotype, and that the level of RTV may depend on the nature of the task.

Contrary to our predictions, only the ASD group showed impairment in motor inhibition on the Go/NoGo task. This is in contrast to other studies that found inhibitory impairment was present in ADHD but not ASD [18–20]. Differences in samples may partly explain disparities; prior work has only included individuals with ASD with IQ > 70, and has used different tasks (e.g., Stroop task). Given prior literature [11, 16], similar to our interpretation of the trend level increase in premature responses in the ADHD group, we suggest that our limited sample size impacted our ability to detect significant differences. Although the ADHD vs. TD contrast was not significant, the directionality of effect was in line with our expectations (i.e., that ADHD were more impaired than TD; *p* = 0.11), and is comparable to other studies that report inhibition impairments in ADHD [11, 12]. Difficulties in inhibition, but also increased premature response as discussed above, in the ASD group, suggests it is important in neuropsychological studies of ADHD populations, to control for co-occurring ASD traits, which are of substantial prevalence [8]. No impairment in motor inhibition was found in the ODD/CD group. It may be that ODD/CD can be differentiated from ADHD and ASD by the nature of inhibition difficulties. Inhibition impairments in ODD/CD are found in more challenging inhibition tasks requiring withholding of an already triggered motor response such as the Stop task [4, 6], but not on tasks of relatively simpler, selective motor response inhibition such as the Go/NoGo task [4].

The lack of group differences in cognitive flexibility is in line with studies that find neither ADHD [12, 18, 21], nor ODD/CD [4] are characterised by such impairments, in particular in easy perceptual switch tasks like the one used in this study. It was unexpected that the ASD group would not show cognitive flexibility impairment given prior research [15]. The use of a relatively simple perceptual switching task may be related to this spared performance, as compared to well-replicated impairments in ASD groups on the more difficult Wisconsin Card Sorting Task that requires content switching and also taps into working memory [15].

Overall, the ASD group showed the most robust EF impairments, specifically in aspects of inhibition. This differs from previous work [18–20], and is most likely due to selection of a more representative group of individuals with ASD (e.g., not limited to IQ > 70, potentially with other co-occurring diagnoses). However, results may not solely be due to these factors, as the findings remained when controlling for IQ and additional ADHD and ODD/CD symptoms, and in sensitivity analyses excluding those with ASD and IQ < 70, and those above a clinically meaningful threshold for ADHD symptoms. Thus, along with previous literature [14, 15], findings suggest ASD is characterised by not only impairments in social, but also in aspects of non-social cognition.

One interpretation of results overall is that ASD is associated with disorder-specific impairments in EF (in premature responding and pre-potent response inhibition); however, we are hesitant to make this claim as although current analyses did not find impairment in the ADHD group, a wealth of literature has demonstrated similar inhibition impairments in ADHD (e.g., [11, 16]). Therefore, we suggest that the current null results are most likely due to power issues associated with small sample sizes, combined with the heterogeneity of EF impairments in ADHD [41]. Instead we propose that further research is required, with larger samples of individuals with ADHD, to clarify the nature of shared impairments between ASD and ADHD. These findings would contribute to the wider debate regarding the validity of our current diagnostic systems, and support the idea that using measurable endophenotypes as indices of cognitive/brain functioning may yield fruitful insights into the aetiology of psychopathology [42].

### Strengths and limitations

To our knowledge this is the first study to directly compare EF among ADHD, ODD/CD and ASD groups. Strengths include accounting for co-occurring ADHD and ODD/CD symptoms, which prior studies (e.g., [18, 19]) have not consistently done, and using a more representative sample of individuals with ASD. One limitation is that, due to the post hoc nature of data analysis, we did not have information on ASD symptoms in the ADHD and ODD/CD groups. Although there is little evidence to suggest increased likelihood of ASD in those with ODD/CD, ASD traits are elevated in those with ADHD [8]. Thus, although the ADHD and ODD/CD group were screened for ASD diagnoses, it is possible that unacknowledged, sub-threshold ASD traits could have impacted upon our findings.

Additionally, unlike for the ODD/CD and ADHD groups, we did not have any formal diagnostic information on co-occurring psychopathology in the ASD group and instead used parent-rated symptoms to identify individuals with high levels of hyperactivity and conduct problems. Whether diagnostic assessments would identify the same individuals as parent-rated questionnaires is an open question. We conducted sensitivity analyses excluding participants with ASD and high rates of ADHD symptoms; however, we were not able to conduct theoretically similar analyses with participants with ASD and high levels of conduct problems due to low group numbers (*n* = 4), and so these participants were excluded from the study completely. The low rates of clinically significant conduct problems in the ASD group are consistent with previous studies of co-occurring psychopathology in ASD [9, 34].

Another potential limitation is that samples were ascertained separately, and thus were mismatched on demographics. However, we controlled for these differences in the analyses. It should also be noted that the current study included modest sample sizes, limiting statistical power to detect differences and thus requires replication.

## Conclusions

Current results suggest that certain EF impairments, such as motor response inhibition, and a premature response style, may not be specific to ADHD, as previous literature has suggested, but are present in other disorders, such as ASD. Whether the cognitive phenotypes associated with diagnostic categories found in the current study represent risk factors requires investigation in longitudinal samples. Results highlight the importance of accounting for additional sub-threshold symptoms, especially those relating to ASD, when comparing different diagnostic groups on measures of cognitive functioning. Disentangling both common and distinct cognitive impairments in terms of diagnostic groups, and the associations between cognitive functioning and specific domains of symptoms, will aid in understanding the underlying mechanisms of different disorders.

## Electronic supplementary material

Below is the link to the electronic supplementary material.

Supplementary material 1 (DOCX 14 kb)
