# Supplementary material for: Testing the specificity of executive functioning impairments in adolescents with ADHD, ODD/CD and ASD
Source: Eur Child Adolesc Psychiatry. 2017 Dec 9;27(7):899–908. doi: 10.1007/s00787-017-1089-5 (PMC6013506; doi:10.1007/s00787-017-1089-5)
Supplement: Supplementary file 1 — Supplementary material 1 (DOCX 14 kb) [file 787_2017_1089_MOESM1_ESM.docx]

*Supplementary Appendix*

|  | Unadjusted post-hoc contrasts | Adjusted  for IQ, age, sex | Adjusted  for ADHD symptoms, IQ, age, sex | Adjusted  for conduct problems, IQ, age, sex |
| --- | --- | --- | --- | --- |
| Go/NoGo: Probability of inhibition | ASD<CD/ODD**, ADHD**, TD** | ASD<CD/ODD**, ADHD**, TD** | ASD<CD/ODD**, ADHD**, TD** | ASD<CD/ODD**, ADHD**, TD** |
| Switch: RT Cost | - | - | - | - |
| Switch: Error Cost | - | - | - | - |
| Go/NoGo: ICV | TD<ADHD**, ODD/CD**, ASD** | TD<ADHD*, ODD/CD*, ASD** | - | - |
| Switch: ICV | TD<ADHD**, ODD/CD**, ASD**  ASD<ODD/CD* | TD<ADHD*, ODD/CD** | - | - |
| Go/NoGo:  Premature responses | ADHD>TD^  ODD/CD>TD*  ASD>TD** | ODD/CD>TD^  ASD>TD** | ASD>TD* | ASD>TD** |
| Switch:  Premature responses | - | - | - | - |

*Un/Adjusted Post-hoc Contrasts of Group Differences Where ANOVA Analyses Found a Significant Effect of Diagnostic Group*

***p*<0.01, **p*<0.05, ^*p*=0.06; ICV intra-individual coefficient of variability; RT reaction time.

*Unadjusted Post-hoc Contrasts of Group Differences Where ANOVA Analyses Found a Significant Effect of Diagnostic Group in Sensitivity Analyses*

|  | Exclude those with IQ<70 | Exclude those in ASD over ADHD cut off |
| --- | --- | --- |
| Go/NoGo: Probability of inhibition | ASD<CD/ODD**, ADHD*, TD** | ASD<CD/ODD**, ADHD*, TD** |
| Switch: RT Cost | - | - |
| Switch: Error Cost | - | - |
| Go/NoGo: ICV | TD<ADHD**, ODD/CD**, ASD** | TD<ADHD**, ODD/CD**, ASD** |
| Switch: ICV | TD<ADHD**, ODD/CD**, ASD^  ASD<ODD/CD^ | TD<ADHD**, ODD/CD** |
| Go/NoGo:  Premature responses | ODD/CD>TD*  ASD>TD** | ADHD>TD^  ODD/CD>TD*  ASD>TD** |
| Switch:  Premature responses | - | - |

***p*<0.01, **p*<0.05, ^*p*=0.06; ICV intra-individual coefficient of variability.
